# MRI radiomics-based decision support tool for a personalized classification of cervical disc degeneration: a two-center study

**DOI:** 10.3389/fphys.2023.1281506

**Published:** 2024-01-03

**Authors:** Jun Xie, Yi Yang, Zekun Jiang, Kerui Zhang, Xiang Zhang, Yuheng Lin, Yiwei Shen, Xuehai Jia, Hao Liu, Shaofen Yang, Yang Jiang, Litai Ma

**Affiliations:** ^1^ Information Technology Center, West China Hospital of Sichuan University, Chengdu, China; ^2^ Information Technology Center, Sanya People’s Hospital, Sanya, China; ^3^ Department of Orthopedics, Orthopedic Research Institute, West China Hospital, Sichuan University, Chengdu, Sichuan, China; ^4^ College of Computer Science, Sichuan University, Chengdu, Sichuan, China; ^5^ West China Biomedical Big Data Center, Sichuan University, Chengdu, Sichuan, China; ^6^ Cadre Health Section, Hezhou People’s Hospital, Hezhou, Guangxi, China; ^7^ Department of Orthopedic Spine, The Second Affiliated Hospital of Chengdu Medical College (China National Nuclear Corporation 416 Hospital), Chengdu, Sichuan, China

**Keywords:** cervical disc degeneration, magnetic resonance imaging, radiomics, machine learning, quantitative image analysis

## Abstract

**Objectives:** To develop and validate an MRI radiomics-based decision support tool for the automated grading of cervical disc degeneration.

**Methods:** The retrospective study included 2,610 cervical disc samples of 435 patients from two hospitals. The cervical magnetic resonance imaging (MRI) analysis of patients confirmed cervical disc degeneration grades using the Pfirrmann grading system. A training set (1,830 samples of 305 patients) and an independent test set (780 samples of 130 patients) were divided for the construction and validation of the machine learning model, respectively. We provided a fine-tuned MedSAM model for automated cervical disc segmentation. Then, we extracted 924 radiomic features from each segmented disc in T1 and T2 MRI modalities. All features were processed and selected using minimum redundancy maximum relevance (mRMR) and multiple machine learning algorithms. Meanwhile, the radiomics models of various machine learning algorithms and MRI images were constructed and compared. Finally, the combined radiomics model was constructed in the training set and validated in the test set. Radiomic feature mapping was provided for auxiliary diagnosis.

**Results:** Of the 2,610 cervical disc samples, 794 (30.4%) were classified as low grade and 1,816 (69.6%) were classified as high grade. The fine-tuned MedSAM model achieved good segmentation performance, with the mean Dice coefficient of 0.93. Higher-order texture features contributed to the dominant force in the diagnostic task (80%). Among various machine learning models, random forest performed better than the other algorithms (*p* < 0.01), and the T2 MRI radiomics model showed better results than T1 MRI in the diagnostic performance (*p* < 0.05). The final combined radiomics model had an area under the receiver operating characteristic curve (AUC) of 0.95, an accuracy of 89.51%, a precision of 87.07%, a recall of 98.83%, and an F1 score of 0.93 in the test set, which were all better than those of other models (*p* < 0.05).

**Conclusion:** The radiomics-based decision support tool using T1 and T2 MRI modalities can be used for cervical disc degeneration grading, facilitating individualized management.

## 1 Introduction

Neck pain is a highly prevalent musculoskeletal condition and the fourth leading cause of disability that has become a serious public health issue worldwide, imposing an enormous burden on patients, healthcare system, and the economic structure of countries (Cohen, 2015; Cohen and Hooten, 2017). According to the Global Burden of Disease 2017 study, the number of prevalent cases of neck pain was 288.7 million, and there has been a substantial increment in the past 3 decades (Safiri et al., 2020). A recent study reported that the annual cost for the treatment of neck pain and low back pain was estimated to be $134.5 billion in the US, ranking first in terms of healthcare spending (Dieleman et al., 2020). Despite its high prevalence and the huge burden imposed on society, it receives relatively less research attention compared to low back pain (Cohen, 2015).

Similar to low back pain, a widely recognized contributor to neck pain is the degeneration of the cervical intervertebral disc (Fujimoto et al., 2012; Risbud and Shapiro, 2014; Theodore, 2020). The intervertebral disc comprises the peripherally located annulus fibrosus (AF), interior gel-like nucleus pulposus (NP), and cartilaginous endplates (CEPs), interposing between the two adjacent vertebral bodies and acting as the shock absorber of the spine. The most physiologically important degenerative changes in the intervertebral disc commence in NP and are usually characterized as decreased water content and loss of disc height, accompanied with the decreased yield strength of AF (Antoniou et al., 1996; Ferrara, 2012). These degenerative changes may alter biomechanical transfer and sensitize the nociceptive nerve fibers in the annulus and nucleus pulposus, which leads to disc herniation, nerve compression, and discogenic pain (Binch et al., 2015; Khan et al., 2017; Theodore, 2020). T2-weighted magnetic resonance imaging (MRI) is the most used imaging modality in the diagnosis of cervical degenerative disc disease (CDDD) due to its superiority in detecting the shape of the intervertebral disc and the water content of NP (Farshad-Amacker et al., 2015). Currently, the most widely used MRI classification system for intervertebral disc degeneration is based on the structure and signal intensity of the disc and the distinction of the nucleus and annulus as proposed by Pfirrmann et al. (2001). While each grade of disc degeneration is clearly defined in this system, it is still a laborious and time-consuming task and highly dependent on the expertise of radiologists and surgeons in clinical practice. The accurate and rapid automated classification of cervical disc degeneration on MRI remains a challenge.

Radiomics and deep learning (DL) approaches have proven to be effective methods for medical image analysis and obtained significant advances in the field of musculoskeletal system disease in recent years (Leung et al., 2020; von Schacky et al., 2020; Huang et al., 2020; Won et al., 2020; Gao et al., 2021; Goedmakers et al., 2021; Bayramoglu et al., 2021; Wang et al., 2022; Hallinan et al., 2021; Niemeyer et al., 2021; Zheng et al., 2022; Abdullah and Rajasekaran, 2022; Gebre et al., 2022). Swiecicki et al. (2021) proposed an automated DL model to evaluate the severity of knee osteoarthritis using knee radiographs according to the Kellgren–Lawrence grading system. Gebre et al. (2022) developed and compared DL models to detect hip osteoarthritis on clinical computer tomography (CT). Hallinan et al. (2021) developed a DL model for the automated detection and classification of central canal, lateral recess, and neural foraminal stenosis in the lumbar spine using sagittal and axial MRI. Several studies reported the feasibility of automated grading of lumbar disc degeneration based on MRI (Gao et al., 2021; Niemeyer et al., 2021; Zheng et al., 2022). Gao et al. (2021) proposed a push–pull regularization strategy to improve the convolutional neural network representation capability for intervertebral disc grading and demonstrated its superior performance. Niemeyer et al. (2021) presented a novel DL-based system for automatically evaluating lumbar disc degeneration according to Pfirrmann grading based on T2-weighted MRI slices, which achieved overall superior reproducibility compared with human interrater. Zheng et al. (2022) developed a segmentation network and a quantitation method to evaluate lumbar intervertebral disc degeneration and calculate the signal intensity and geometric features of disc degeneration. However, there is still a paucity of studies reporting the automated classification of cervical disc degeneration on MRI. The much smaller size of the cervical intervertebral disc compared to the lumbar disc and various anatomical morphologies and MRI signal intensities may increase the difficulty of automatic classification of cervical disc degeneration. The Segment Anything Model (SAM), as a vision foundation model, has shown significant advantages in zero-shot and few-shot segmentations in medical imaging (Cheng et al., 2023; Kirillov et al., 2023; Shi et al., 2023), and many studies have embedded it into the development process (Ma et al., 2023; Mazurowski et al., 2023). Zero-shot or few-shot learning implies that with no or a small amount of data, it is possible to use or fine-tune the foundation model to perform exceptionally well in specific downstream tasks. This addresses real-world challenges like limited clinical data or a lack of annotated data. Here, we can consider using the SAM-based DL model to achieve rapid cervical disc segmentation and develop classification diagnosis algorithms on this basis.

Therefore, in this study, we aimed to develop and validate an MRI-based radiomics decision support tool for a personalized classification of cervical intervertebral disc degeneration according to the Pfirrmann scheme (Pfirrmann et al., 2001). To the best of our knowledge, this is the first study to develop the radiomics-based automated classification system for the intervertebral disc degeneration of the cervical spine. The imaging difference of discs with different degeneration grading scores may contribute to the understanding of the mechanisms underlying the onset and progression of disc diseases.

## 2 Materials and methods

### 2.1 Ethics and study design

The study was conducted in accordance with the Declaration of Helsinki (as revised in 2013). This retrospective study was approved by the Ethics Committee of Biomedical Research, West China Hospital (2021-1490). Written informed consent was waived owing to the retrospective nature of data collection (age/gender) and the use of de-identified MRI images.

**FIGURE 1:**
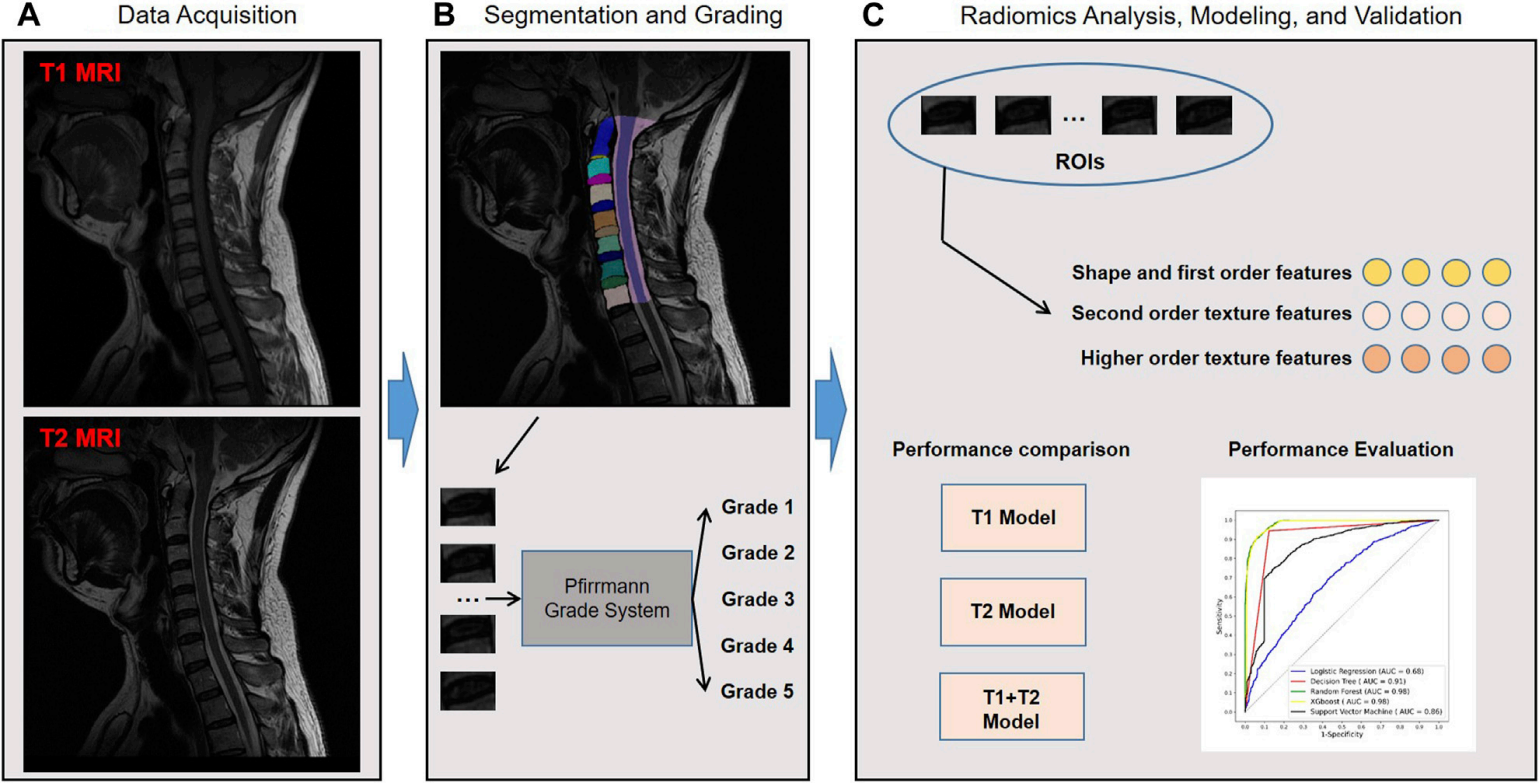
Study workflow overview. Workflow includes **(A)** data acquisition, **(B)** segmentation and grading, and **(C)** radiomics analysis, modeling, and validation. MRI, magnetic resonance imaging; ROIs, regions of interest; AUC, area under the receiver operating characteristic curve; XGBoost, eXtreme Gradient Boosting.

Figure 1 shows the workflow of the study. A total of 2,610 cervical disc samples of 435 patients were retrospectively analyzed. Each patient underwent both T1 and T2 MRI modalities. Each patient had six intercalated discs (C2/3, C3/4, C4/5, C5/6, C6/7, and C7/T1) on the image, which were all semi-automatically segmented using a deep learning-embedded segmentation tool. Then, a fine-tuned MedSAM model was developed for automated disc segmentation. Meanwhile, the grading classification of cervical disc degeneration was performed using the Pfirrmann grading system. Based on the segmented regions of interest (ROIs), i.e., every disc, we extracted the high-throughput radiomic features including shape and first-order features, second-order texture features, and higher-order texture features. Then, we performed the diagnosis performance comparison between different machine learning algorithms and MRI images. Finally, through comparison, the optimal combined radiomics model was developed in the training set and validated in the test set.

### 2.2 Study population

**FIGURE 2:**
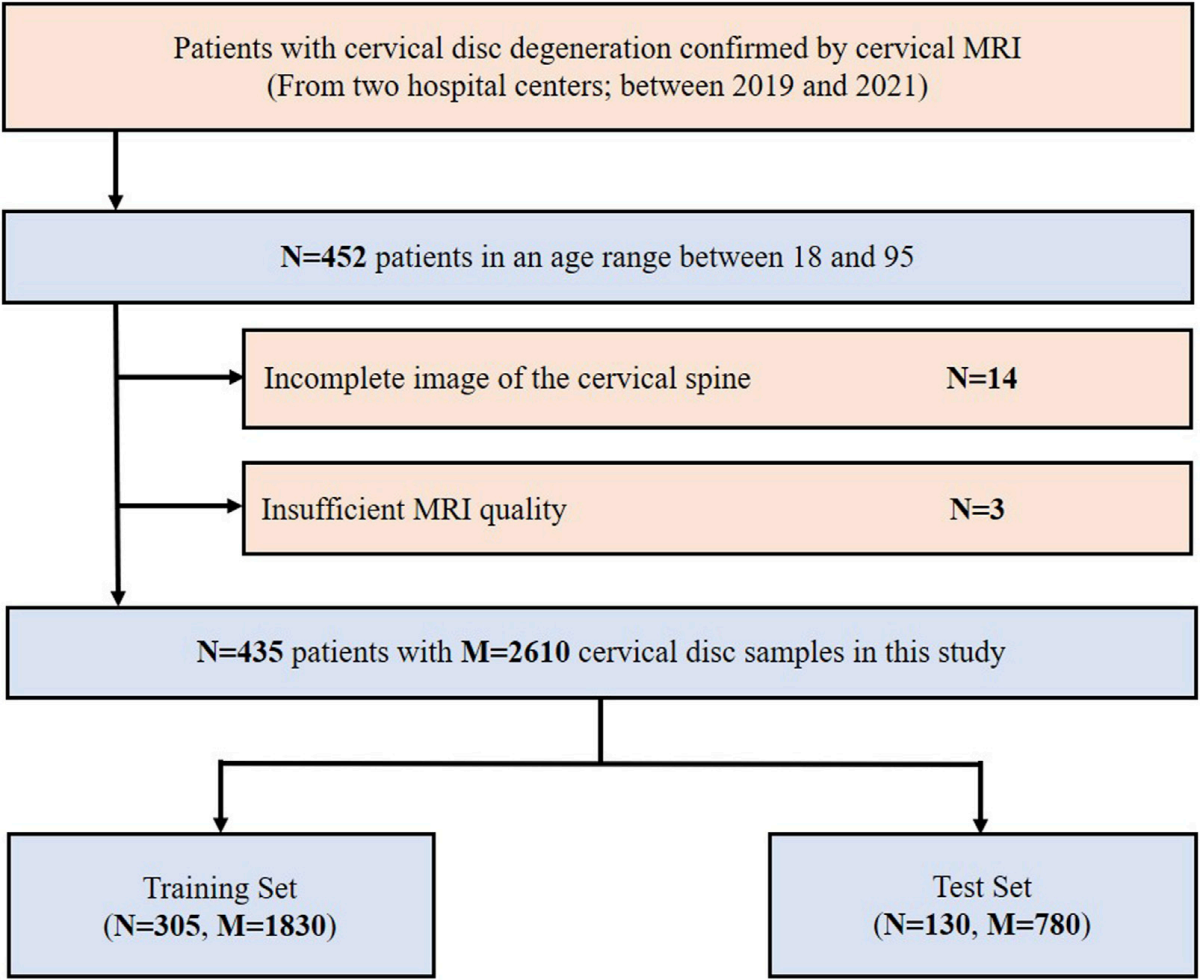
Inclusion flowchart of the study population. MRI, magnetic resonance imaging; N, the number of patients; M, the number of cervical discs.

A total of 452 consecutive patients aged between 18 and 95, for whom cervical MRI was prescribed for medical reasons, were scanned between 2019 and 2021 at the West China Hospital of Sichuan University and the First People’s Hospital of Longquanyi District using either Siemens 3.0T scanners. Overall, 17 patients were excluded for the following reasons: 1) incomplete image of the cervical spine (*n* = 14) and 2) insufficient MRI quality (*n* = 3). Finally, 435 patients were retrospectively collected in this study. The inclusion and exclusion flowchart of the study population is shown in Figure 2.

The data were randomly divided into a training set (1,830 study samples of 305 patients) and a test set (780 study samples of 130 patients) according to the ratio of 7:3. The radiomics analysis, feature selection, and model development were implemented in the training set, and then the related radiomics models were validated in the test set.

### 2.3 MRI data acquisition

All MRI examinations were performed on a 3.0T MRI system (MAGNETOM Skyra, Siemens, Germany and Discovery 750w, GE, United States) with a phased-array body surface coil. The imaging protocol was the same for all patients. Images were acquired with a sagittal T2-weighted spin echo sequence with the following parameters: repetition time (TR), 3,064–6,220/3,675–4,200 msec; echo time (TE), 102–104/74 msec; matrix, 288 × 224/128 × 128; field of view (FOV), 256 × 256 mm^2^; slice thickness, 3.6/3.6 mm; and intersection gap, 2 mm. T1-weighted MRI was acquired with the following parameters: an inversion time of approximately 400 ms, resolution at 0.8*0.8*0.4 mm, 25 slices in 3 min 20 s, and single-shot mode with an echo train length (ETL) of 230.

### 2.4 Fine-tuned MedSAM segmentation model

We segmented the cervical discs from T1 and T2 MRI modalities separately. Six discs (C2/3, C3/4, C4/5, C5/6, C6/7, and C7/T1) were segmented as the independent study samples. First, ROI segmentation was performed by two orthopedic radiologists with approximately 5 years of experience by using Pair software (https://www.aipair.com.cn/en/, Version 2.7, RayShape, Shenzhen, China), which embedded deep learning algorithms and trained segmentation models inside (Liang et al., 2022). The final segmentation results were checked and modified by a third radiologist with more than 10 years of experience. By using the DL-based segmentation tool, the work efficiency had been significantly improved.

For achieving fast automated segmentation of discs, we provided a disc segmentation model by fine-tuning the MedSAM model (Ma et al., 2023) based on real results. The image encoder and box prompt encoder were frozen, and the mask encoder was re-trained for the task. Here, we only fine-tuned the MedSAM model on 64 MRI data with real segmentation, and the fine-tuned MedSAM model was evaluated in other datasets. The Dice coefficient was used to evaluate the segmentation performance.

### 2.5 Disc degeneration grade assessment

The disc degeneration grade assessment was assessed by an experienced orthopedic radiologist, according to the Pfirrmann guideline, conducted by Pfirrmann et al. (2001). The grading system was performed on T2 MRI, and all the discs were classified into five grades (grade 1–5). To facilitate clinical portable use and promote clinical decision-making, we classified grades 1 and 2 as low-grade disc degeneration and grades 3, 4, and 5 as high-grade (Pfirrmann et al., 2001). Based on the gold standard grading results, the radiomics models were developed using supervised learning in the subsequent experiments.

### 2.6 Radiomics analysis, modeling, and validation

Radiomics analysis was implemented using the PyRadiomics library (https://www.radiomics.io/pyradiomics.html, version 3.0.1), which was a commonly used tool for radiomics development (van Griethuysen JJM et al., 2017). The image preprocessing setting followed the previous work (Dong et al., 2022). Then, a total of 924 radiomic features were quantified, including shape and first-order features (*n* = 32), second-order texture features (*n* = 73), and higher-order texture features (*n* = 819). The second-order texture features were calculated using the gray-level co-occurrence matrix (glcm), gray-level run-length matrix (glrlm), gray-level size-zone matrix (glszm), gray-level dependence matrix (gldm), and neighboring gray-tone difference matrix (ngtdm). The higher-order texture features were quantified using the Laplacian of Gaussian (LoG) and wavelet transformation. The details of radiomic features can be seen in PyRadiomics documentation (https://pyradiomics.readthedocs.io/en/latest/index.html). Most of them follow the image biomarker standardization initiative (IBSI).

For the important feature selection, minimum redundancy maximum relevance (mRMR) was performed, which was a minimal-optimal feature selection method for finding the smallest relevant feature subset (Zhao et al., 2019). mRMR was used for feature selection in many radiomics studies in recent years (Xie et al., 2021; Hou et al., 2022; Yang et al., 2022).

Here, we defined a machine learning pipeline for model construction and selection. Through mRMR feature selection, the top 10 features were selected for further modeling. For imbalance processing, an adaptive synthetic (ADASYN) algorithm, as a valuable oversampled method in radiomics (Han et al., 2023), was performed to balance the training data. Then, the logistic regression, decision tree, random forest, eXtreme Gradient Boosting (XGBoost), and support vector machine (SVM) were implemented for model construction and comparison. Here, all the machine learning hyperparameter optimization (HPO) was performed using Bayesian optimization, which was the state-of-the-art HPO algorithm (Yang and Shami, 2020). All the models were built in the training set and evaluated in the test set. The performance difference between different machine learning models was evaluated in all sets.

By using the machine learning pipeline, the optimal radiomics models were confirmed on T1 and T2 MRI modalities. Finally, we combined the selected T1 and T2 radiomic features and built the combined radiomics model in the training set. The final radiomics model was validated in the test set.

### 2.7 Statistical analysis

All statistical analyses and machine learning algorithms were performed using SPSS (version 25; IBM Corporation) and Python (version 3.8). The Mann–Whitney U test and chi-squared test were implemented for continuous and count variables, respectively. The diagnostic performance was evaluated using the receiver operating characteristic (ROC) curve and AUC. The difference between machine learning models was evaluated using the DeLong test. *p*-value <0.05 was considered significantly different.

## 3 Results

### 3.1 Clinical characteristics

The clinical characteristics of the study population in the training and test sets are shown in Table 1. There was no significant difference in the two datasets. In the training set, 1,303 (71.2%) were defined as the high-grade disc degeneration. Of the test set, 513 (65.8%) disc samples were defined as high grade.

**TABLE 1:** Clinical characteristics of the study population.

Characteristic	Full (N = 435)	Training set (*N* = 305)	Test set (*N* = 130)
Age (years)			
Mean ±	47.66	47.63	47.74
SD	12.87	12.91	12.79
Gender			
Male	298 (68.5%)	213 (69.8%)	85 (65.4%)
Female	137 (31.5%)	92 (30.2%)	45 (34.6%)
Cervical disc samples	2,610	1,830	780
Degeneration grading			
Low grade	794 (30.4%)	527 (28.8%)	267 (34.2%)
High grade	1,816 (69.6%)	1,303 (71.2%)	513 (65.8%)

SD, standard deviation.

### 3.2 MedSAM segmentation

**FIGURE 3:**
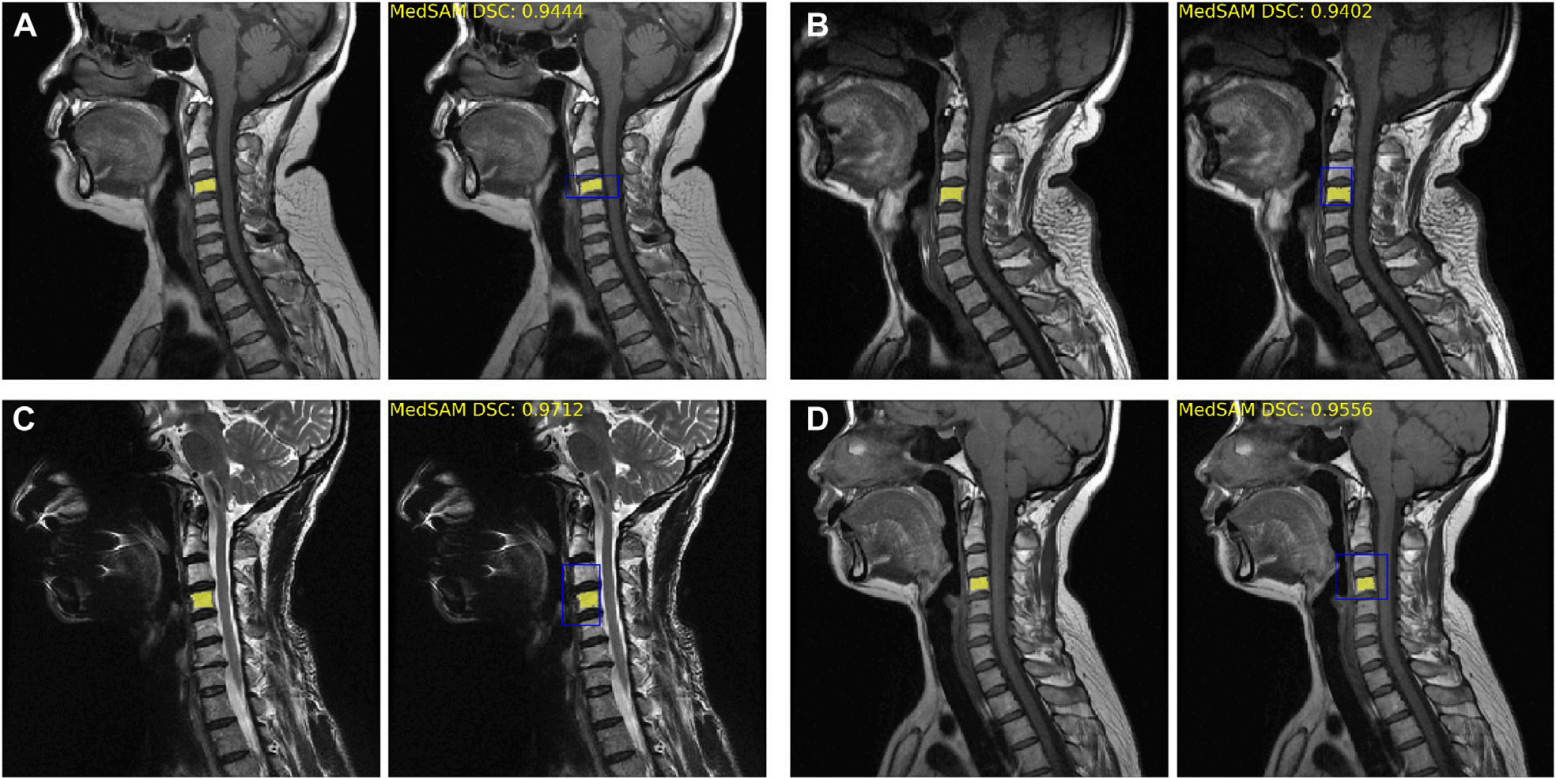
Fine-tuned MedSAM segmentation results. **(A–D)** Four disc segmentation examples. The left side is the real segmentation manually labeled by the doctors, and the right side is the prediction results obtained using MedSAM. The blue bounding box indicates the prompt input, and the yellow area indicates the segmentation results. DSC, Dice similarity coefficient.

The fine-tuned MedSAM count achieved the mean Dice coefficient of 0.93 ± 0.04. Figure 3 provides four examples of MedSAM segmentation, which all showed the good segmentation performance. When using the fine-tuned MedSAM model, the doctor only needs to roughly give a bounding box (like the blue box), and our model can achieve accurate disc segmentation (the yellow area).

### 3.3 Radiomic feature discovery

Table 2 shows the top 10 radiomic features in the T1 and T2 MRI modalities. All the features were selected using mRMR. Most of them were higher-order texture features (*N* = 16, 80%), including LoG and wavelet transform features. Of the feature type, first-order (*N* = 8, 40%) and glcm (*N* = 4, 20%) were the dominant factors. Compared with T1 and T2 radiomic features, we observe that the key features were relatively similar. In particular, the kurtosis feature appeared three times, and the kurtosis of log-sigma-5.0-mm first-order feature was the highest ranked radiomic feature in both MRI modalities.

**TABLE 2:** Top 10 radiomic features on T1 and T2 MRI modalities.

MRI	Transformation	Feature type	Radiomic feature
T1	log-sigma-5-0-mm	First-order	Kurtosis
	wavelet-HH	ngtdm	Busyness
	original	First-order	10 percentile
	wavelet-HH	glcm	IMC1
	log-sigma-5-0-mm	First-order	Minimum
	log-sigma-1-0-mm	First-order	Skewness
	log-sigma-4-0-mm	First-order	10 percentile
	original	Shape	Maximum 2D Diameter Row
	wavelet-HH	glrlm	Run variance
	wavelet-LL	First-order	Kurtosis
T2	log-sigma-5-0-mm	First-order	Kurtosis
	log-sigma-2-0-mm	glszm	Gray-level non-uniformity
	wavelet-LL	glcm	Cluster prominence
	original	glszm	Large area low gray-level emphasis
	wavelet-HL	glcm	IMC1
	log-sigma-2-0-mm	First-order	Skewness
	original	Shape	Maximum 2D diameter column
	wavelet-LL	glcm	Correlation
	log-sigma-3-0-mm	glrlm	Long run low gray-level emphasis
	log-sigma-5-0-mm	gldm	Dependence variance

Log, Laplacian of Gaussian; ngtdm, neighboring gray-tone difference matrix; glcm, gray-level co-occurrence matrix; glrlm, gray-level run-length matrix; glszm, gray-level size-zone matrix; gldm, gray-level dependence matrix; IMC1, informational measure of correlation 1.

### 3.4 Diagnostic performance across T1 and T2 MRI modalities

**FIGURE 4:**
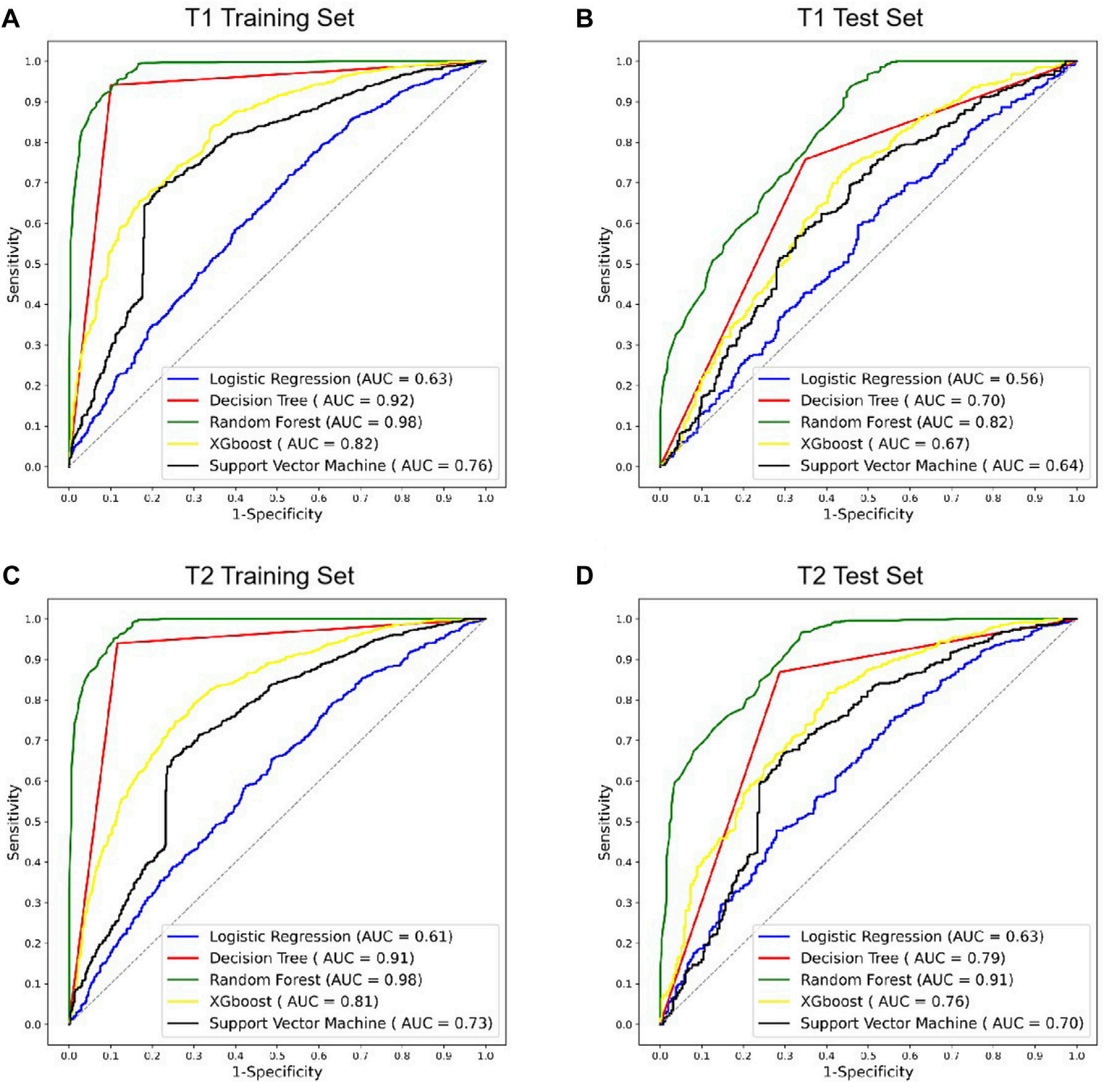
ROC curves of different radiomics models in T1 and T2 MRI modalities. **(A,B)** ROC curves of radiomics models using T1 MRI in the training and test sets. **(C,D)** ROC curves of radiomics models using T2 MRI in the training and test sets. ROC, receiver operating characteristic; AUC, area under the ROC curve; XGBoost, eXtreme Gradient Boosting.

The radiomics models of various machine learning algorithms and MRI images were constructed and compared (Figure 4). In all the modes, random forest obtained the higher diagnostic performance than other machine learning algorithms (AUC = 0.82, *p* < 0.01, T1 test set; AUC = 0.91, *p* < 0.01, T2 test set). The performance of T2 MRI was significantly higher than T1 MRI (*p* < 0.05).

### 3.5 Diagnostic performance of the combined radiomics model

**FIGURE 5:**
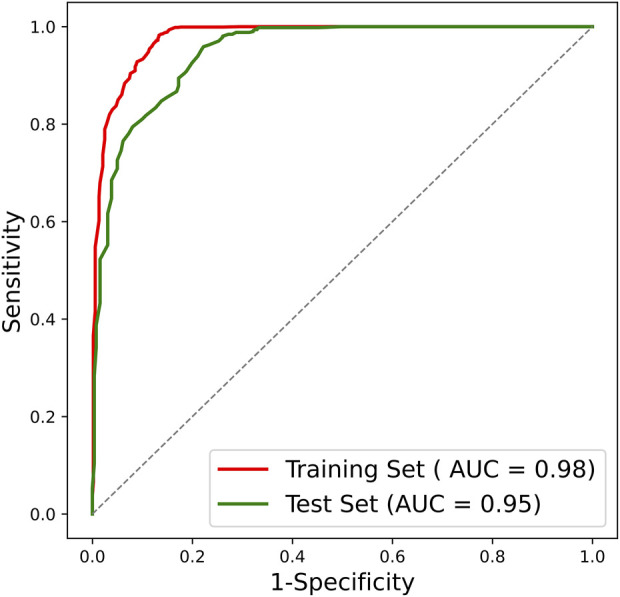
ROC curve of the combined radiomics model. ROC, receiver operating characteristic; AUC, area under the ROC curve.

After confirming the selected radiomic features and random forest modeling method, the final combined radiomics model was constructed and evaluated. Figure 5 shows the ROC curves of the combined model. The AUC of the training ROC was 0.98 and the test AUC was 0.95. Table 3 shows the diagnostic performance between the final T1, T2, and combined radiomics models. The combined model had the accuracy of 89.51%, the precision of 87.07%, the recall of 98.83%, and the F1 score of 0.93 in the test set, which was better than those of other models (*p* < 0.05).

**TABLE 3:** Diagnosis performance of the final radiomics models.

Model	Training set				Test set			
	Accuracy (%)	Precision (%)	Recall (%)	F1	Accuracy (%)	Precision (%)	Recall (%)	F1
T1 MRI	94.95	94.07	99.15	0.96	80.72	79.13	96.28	0.87
T2 MRI	93.96	93.73	98.07	0.95	85.89	83.75	97.66	0.90
Combined	94.94	94.38	98.76	0.97	89.51	87.07	98.83	0.93

### 3.6 Radiomic feature mapping

**FIGURE 6:**
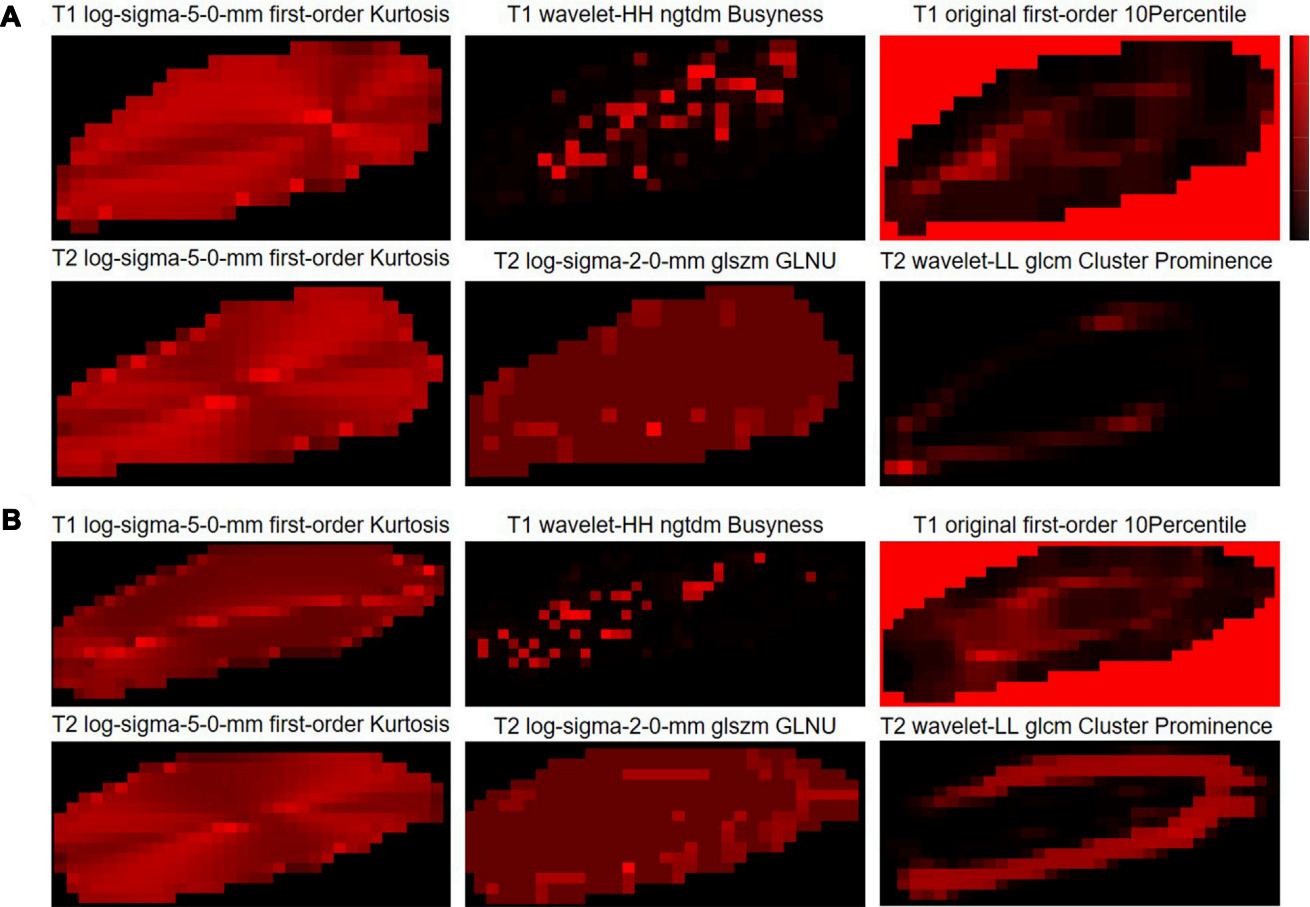
Radiomic feature maps for the classification of cervical disc degeneration. **(A)** Low-grade cervical disc and **(B)** high-grade cervical disc. Log, Laplacian of Gaussian; ngtdm, neighboring gray-tone difference matrix; glszm, gray-level size-zone matrix; GLNU, gray-level non-uniformity; glcm, gray-level co-occurrence matrix.

Here, in order to provide the visualization tools that are easy to use clinically, the radiomic feature maps are provided (Figure 6). Only the top three feature maps of the two modalities were used to aid in diagnosis, enabling some mode differences to be seen on these virtual medical imaging through the comparison of low- and high-grade disc degeneration.

## 4 Discussion

There is still a paucity of studies reporting the automated grading of cervical disc degeneration on MRI. In clinical practice, the automated decision support tool to intelligently grade disc degeneration through MRI will greatly assist physicians in individualized patient management. In this study, we developed and validated an MRI radiomics-based cervical disc degeneration grading method, which can automatically segment the disc ROIs, extract valuable radiomic features, and predict the degeneration grades, showing a high diagnostic performance (AUC = 0.98 in the training set; AUC = 0.95 in the test set).

We found that both T1 and T2 MRI modalities showed good diagnostic results, although T2 showed higher performance than T1. To the best of our knowledge, T2 MRI is the most used MRI modality in the diagnosis of cervical degenerative disc disease (Farshad-Amacker et al., 2015), and many studies developed AI models only based on T2 MRI (Gao et al., 2021; Zheng et al., 2022). Our study showed that even though T1 MRI was macroscopically difficult to use directly for disc grading, it was still possible to construct the diagnostic model with good performance through radiomics and machine learning methods. By integrating the valuable information in T1 and T2, the performance of the combined model will significantly be improved, which may have some positive hints for clinical practice.

For the valuable radiomic features, higher-order texture features showed the dominant force, which was consistent with the findings of previous studies (Jiang et al., 2022a; Jiang et al., 2022b). Texture features can adequately characterize the heterogeneous information between tumors or inflammation (Alobaidli et al., 2014; Song et al., 2023), and the higher-order transformations (wavelet, LoG, convolutional neural network, etc.) may have the potential to further enhance the expression of this heterogeneity, contributing to the diagnostic performance (Jiang et al., 2022a; Jiang et al., 2022b). The mRMR, ADASYN, and Bayesian optimization algorithms also showed the excellent selection performance in radiomics analysis, which is consistent with the findings of Xie et al. (2021), Hou et al. (2022), Wang et al. (2023), and Wu et al. (2023).

Here, we also provided the radiomic feature maps for the classification of cervical disc degeneration (Figure 5). We can still visualize relatively well the differences in patterns between low- and high-grade degeneration, especially the log-sigma-5.0-mm first-order kurtosis. However, in fact, the difference in this virtual imaging was not very significant, and it was still difficult to be used as an independent imaging biomarker that gives clinicians a direct and significant indication. However, to a certain extent, the imaging difference of discs with different degeneration grading scores may help understand the mechanisms underlying the onset and progression of disc diseases.

In the study, we used each cervical disc as an independent study sample, and all six different cervical discs in each person were mixed to perform the modeling analysis. The better diagnostic performance showed that the different cervical discs could be identified basically using the same mode. In addition, the gold standard for Pfirrmann assessment usually classifies discs into five categories, and for the purpose of clinical decision-making we have used only two classifications: low grade (grades 1 and 2) and high grade (grades 3, 4, and 5). In future studies, we will further expand the amount of data to build clinical tools for automatic segmentation and five-category diagnosis. Moreover, we will also consider other clinical grading criteria (Adams and Dolan, 2012; Wáng, 2018) and compare the performance differences between machine learning models built under different criteria.

There are also some limitations to the study. First, as a retrospective study, there was no clinical information enrolled. Perhaps adding the broader range of clinical factors could further enhance the performance of the model. Second, although it was a two-center study, there was still a need to further expand the validation of independent center data for the decision support tool to be further promoted and validated. Therefore, a larger multi-center study is needed.

## 5 Conclusion

In conclusion, we demonstrated that the radiomics-based decision support tool by integrating T1 and T2 MRI modalities can be used for a personalized classification of cervical disc degeneration, showing the robust diagnostic performance, and may aid in clinical decision-making and individualized management.

## Data Availability

The original contributions presented in the study are included in the article/Supplementary Material; further inquiries can be directed to the corresponding authors.

## Abbreviations

MRI, magnetic resonance imaging; ROC, receiver operating characteristic; AUC, area under the curve; AF, annulus fibrosus; NP, nucleus pulposus; CDDD, cervical degenerative disc disease; DL, deep learning; CT, computed tomography; glcm, gray-level co-occurrence matrix; glrlm, gray-level run-length matrix; glszm, gray-level size-zone matrix; gldm, gray-level dependence matrix; ngtdm, neighboring gray-tone difference matrix; LoG, Laplacian of Gaussian; mRMR, minimum redundancy maximum relevance; XGBoost, eXtreme Gradient Boosting; SVM, support vector machine.
